# Greater patient access to immuno-oncology therapies—what can policymakers do?

**DOI:** 10.3332/ecancer.2015.ed48

**Published:** 2015-02-25

**Authors:** Francesco de Lorenzo, Suzanne Wait, Burçak Karaca, Cedrik M Britten, Marc van den Bulcke, The European Expert Group on Immuno-Oncology

**Affiliations:** 1 European Cancer Patient Coalition, Brussels B-1000, Belgium; 2 The Health Policy Partnership, London W1W 5NA, UK; 3 Ege University, Department of Medical Oncology, Izmir 35100, Turkey; 4 Association for Cancer Immunotherapy (CIMT), Mainz D-55116, Germany; 5 Scientific Institute of Public Health, B-1050, Belgium; * *Members of the European Expert Group on Immuno-Oncology include: Francesco de Lorenzo (European Cancer Patient Coalition (ECPC)); Philippe de Backer, MEP; Cristian Silviu Busoi, MEP; Cedrik M. Britten (Association for Cancer Immunotherapy (CIMT); Marc van den Bulcke (Institute of Public Health, Belgium); Szymon Chrostowski (Let’s Win Health Foundation, Poland); Edith Frenoy (European Federation of the Pharmaceutical Industries and Associations); Christoph Huber (CIMT); Burçak Karaca (Ege University, Department of Medical Oncology, Izmir); James Larkin (Royal Marsden Hospital, UK); Cilia Linssen (Lung Cancer Europe (LUCE)); Olivier Michielin (European Society for Medical Oncology (ESMO)); Mihaela Militaru (ECPC); Ingrid van den Neucker (European Cancer Coalition (ECCO)); Francisco Ventura Ramos (Portuguese Institute of Oncology, Lisbon).*

Scientists have been looking at the role the immune system may play in fighting cancer for decades, but it is only recently that this knowledge has been translated into immuno-oncology therapies that provide the promise of long-term quality survival to patients [1–3]. Agents are now available to patients with advanced melanoma [1] and castration resistant prostate cancer, [3] and many more agents are expected to become available in the next few years, including in some of most common (e.g. breast and lung) and most difficult-to-treat cancers [1–5].

What makes these new therapies unique is that they confer long-lasting memory to the immune system, enabling it to adapt itself to fight against cancer cells even after remission. Side-effects also tend to be manageable compared to many other cancer therapies.

But as with all new treatments, providing fast access to immuno-oncology therapies to appropriate patients will be increasingly challenging in our resource-constrained health care systems. In addition, the science of immuno-oncology is rapidly evolving, and we are still learning how to use these agents to achieve the best possible response in patients [4]. We still lack a clear understanding of what biological factors make a given immuno-oncology therapy work in a particular patient [6]. Also, the clinical development pathways traditionally used in oncology were typically developed for conventional cytotoxic therapies and may be poorly suited to reflect the effects seen with immunooncology therapies [7].

With these challenges in mind, the European Expert Group on Immuno-Oncology was established in early 2014 as an independent network of patients, oncology healthcare professionals, scientists, industry and politicians to help improve overall understanding of what immuno-oncology therapies are and what they may mean for cancer patients.

A first goal of the Expert Group was to help policymakers identify what policy adaptations were needed to enable rapid access to immunooncology therapies in appropriate patients across Europe. The resulting Policy Action Framework [8] was launched at the European Parliament in November 2014 along with a guide for patients on immuno-oncology [9].

The Action Framework proposes five priority areas, as shown in Figure 1.

Promoting a greater understanding of immuno-oncology among policymakers, regulatory and Health Technology Assessment (HTA) agencies, health professionals and patients is key. Informed policymakers are more likely to integrate new therapies into cancer plans and policies, and ensure that appropriate resources and funding are put in place to deliver on these plans at the local level. Professional training across the entire care team is essential for treatments to be used as appropriately as possible in patients who are most likely to benefit, and to monitor the long-term effects, including toxicity, of this new group of agents. And patients need continuous information and support in order to engage fully in treatment decisions.

Long-term quality survival is the ultimate goal of cancer therapy [10], however obtaining this evidence may take several years. Therefore, it is important to identify validated intermediary endpoints that are predictive of long-term survival [11]. Integrating appropriate measures of quality of life into clinical trials and clinical practice is also critical to assess the impact of new therapies on patients both during and after treatment. Also, HTA agencies and subsequent funding decisions should be based on what matters most to patients and their families, and take account of the full societal benefits of new therapies, for example individuals’ ability to return to work and lead a ‘normal’ life with long-term survival.

Greater flexibility is also needed in existing regulatory frameworks to allow for an appropriate balance between the need for safety and efficacy data and rapid access to promising therapies [12]. For example, the European Medicines Agency is currently exploring more flexible frameworks which allow regulatory decisions to be based on an evolving set of data for selected therapies [13, 14] Early collaboration between drug developers, patient organisations, regulatory agencies and HTA agencies is instrumental for the success of such schemes, as they need to agree to a comprehensive development and licensing plan early on, matched with adaptive frameworks for reimbursement and HTA decisions as well.

Collaborative, multidisciplinary research platforms in the form of public-private partnerships (PPPs) also have a huge role to play in immuno-oncology. For example, the Innovative Medicines Initiative is Europe’s largest PPP aimed at speeding up the development of, and patient access to, innovative medicines, particularly in areas where there is high unmet patient need [15]. PPPs may also help provide access to immuno-oncology therapies in poorer-resourced countries as part of compassionate use programmes. Collaborations between the research community and pharmaceutical companies may also allow the creation of large databases that combine data from several clinical trials. These data may help identify which patient characteristics make individuals more likely to respond to different immuno-oncology therapies and develop suitable biomarkers and other patient selection tools as a result.

Finally, mechanisms need to be put in place to make sure that the most effective new therapies are included in local care pathways and supported by appropriate funding streams, particularly in heavily decentralised health care systems. For example, the Melanoma Taskforce in the UK developed a national toolkit for local commissioners responsible for funding care to ensure equitable, timely and appropriate access to the full range of approved treatments for people with advanced melanoma across all localities. Long-term alternative financing mechanisms that address payer concerns and maximize and accelerate patient access will also be critical.

## Conclusions

In conclusion, the Policy Action Framework is intended as a starting point to building a sustainable and enabling policy environment for the introduction of immuno-oncology therapies in Europe. The challenge now is implementation. This will require close collaboration across borders as well as between all key stakeholder groups - patients, professionals, regulators, policymakers, HTA authorities, payers, and the biomedical industry.

## Figures and Tables

**Figure 1.**
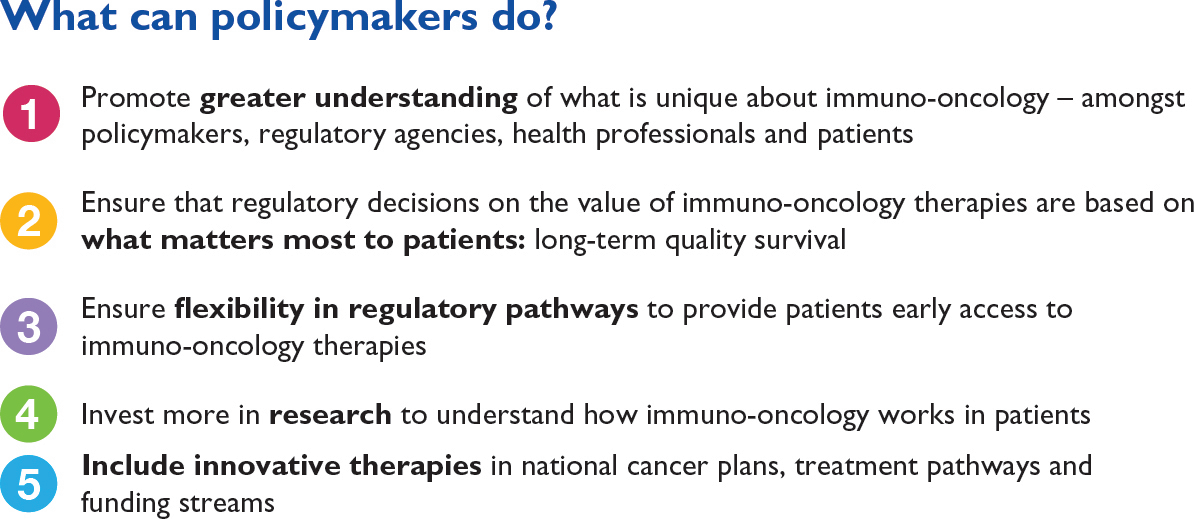
Key recommendations for policymakers on immuno-oncology [8].
